# Editorial: Quinoline as lead structures for the development of leishmanicidal agents

**DOI:** 10.3389/fchem.2025.1694616

**Published:** 2025-09-09

**Authors:** Angel H. Romero, Gustavo Benaim

**Affiliations:** 1 Grupo de Química Orgánica Medicinal, Facultad de Ciencias, Universidad de la República, Montevideo, Uruguay; 2 Unidad de Señalización Celular y Bioquímica de Parásitos, Instituto de Estudios Avanzados (IDEA), Caracas, Venezuela; 3 Instituto de Biología Experimental, Facultad de Ciencias, Universidad Central de Venezuela, Caracas, Venezuela

**Keywords:** quinoline, leishmania, synthesis, nanoencapsulation, structure- activity relationship, antimalarials re-purposing, natural products, phagolysosome

Leishmaniasis comprises a group of neglected diseases present in tropical and subtropical areas caused by the *Leishmania* parasite. It affects approximately 12 million people annually, making it the third most common parasitic condition worldwide (World Health Organization, 2023). Treatment options are limited because there are no vaccines or effective drugs to eradicate the parasites. The drugs currently used present several disadvantages in terms of toxicity, cost, low therapeutic efficacy, prolonged administration treatment times and parasite resistance (Aronson et al., 2016). Moreover, the development of leishmanicidal drugs remains a significant challenge due to rigorous pharmacological requirements and the high genetic plasticity of the parasites. This situation requires the development of new drug discovery strategies that focus not only on classic medicinal chemistry concepts, but also on critical aspects of parasite survival within host cells (Alvar and Arana, 2018). In this context, quinoline emerges as a potential scaffold acting on some key targets of parasite survival, including: (i) altering respiratory complex III, which leads to apoptosis; (ii) enhancing glycolytic ATP synthesis via a sterol-dependent diffusion process; (iii) depolarizing parasite mitochondrial membranes; (iv) accumulating in the parasite’s lipid bodies and, (v) immunostimulating host-cells (Romero and Delgado). Furthermore, the potential of quinoline as a platform for generating potent and selective leishmanicidal agents has been widely demonstrated (Gutierrez et al., 2023; Silva et al., 2024; Valdivieso et al., 2018). As a consequence of the versatility of quinoline as a scaffold, the current Research Topic presents a Research Topic of eight articles. Through reviews, minireviews, and opinion articles, the following Research Topic are discussed: i) the role of the substitution site in the leishmanicidal activity of single quinolines and ii) the potential of natural products, metallic complexes and antimalarial quinoline-based agents as leishmanicidal agents. These discussions highlight mechanistic aspects, structure-activity relationships, *in vivo* results and future perspectives. The synthetic aspects, the role of nanotechnology in improving the leishmanicidal efficacy of quinolines and the importance of lipophilicity/acidity are also discussed.

**FIGURE 1:**
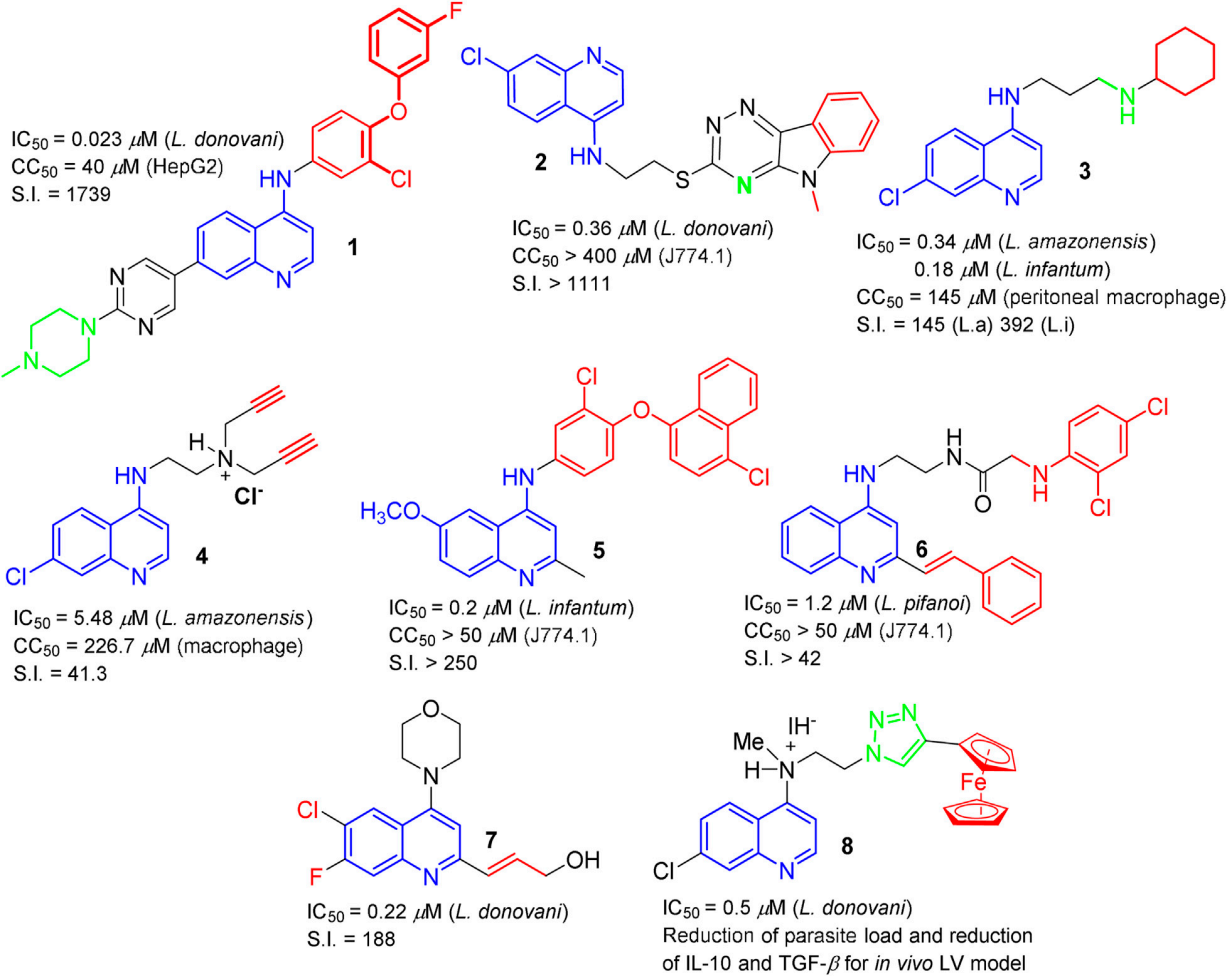
Promising quinoline candidates for preclinical studies.

From the study of 4-aminoquinolines, a privileged scaffold (Romero and Delgado), it was concluded that the inclusion of a basic moiety (preferably a tertiary amine) and lipophilic moieties is essential to increasing the potency and selectivity of quinoline compounds. The substituted aniline moiety at the 4-position was found to be preferred over the amine-alkyl chain. In general, some 4-aminoquinolines have been found to promote depolarization of the parasite’s mitochondrial potential, inducing cell apoptosis and morphological changes. The production of nitric oxide and an increase of proinflammatory cytokines in the infected macrophage models suggest an immunostimulant response to 4-aminoquinoline compounds against intracellular amastigotes. Promising candidates **1-6** for preclinical studies are shown in Figure 1.

Regarding the role of 2-, 3-, 6- and 8-substitution into quinolines (García Liñares and Elso), it was observed that the inclusion of lipophilic chains, such as alkenyl or aryl at the 2-, 3- and 6-positions significantly increased the activity and selectivity in quinoline core (**7**, Figure 1), whereas *O*- or *N*-substitution was preferred at the 8-position. *N*-substitution at the 2 and 3-positions generated compounds with a discrete leishmanicidal response.

From the metallic complexes quinoline-based (Del Carpio et al.), there three types of strutures with leishmanicidal response: i) coordination complexes of Sb(V), Ga(III) or V(IV/V) using 8-quinolinols as a ligand; ii) chloroquine or primaquine analogs bearing a ferrocenyl moiety at the 4-dialkyldiamine chain and; iii) quinolines bearing a metal-NHC moiety. A water-soluble, selective and potent compound against *in vitro* and *in vivo* models of LV was identified among the quinolines bearing a ferrocenyl moiety (**8**, Figure 1). The chloroquine and primaquine nuclei were useful to generate active and selective quinoline-ferrocenyl leishmanicidal compounds. These quinoline-ferrocenyl compounds were found to promote oxidative stress, morphological changes and early apoptosis in the parasite and immunostimulation in infected models. Meanwhile Sb/Ga/V complexes showed a selectivity index (S.I.) lower than 15, whereas NHC-based quinolines were highly toxic with low selectivity (S.I. ˂5).

Regarding natural products (Yaluff et al.), a few examples were found in the literature about the leishmanicidal response of natural quinolines. The majority of these examples focus on 2-alkylquinolines. The inclusion of either an alkyl chain or an aryl chain was found to be key to generating active and potent leishmanicidal agents. This finding further supports the tendency found in the 2-substituted quinolines of synthetic origin (García Liñares and Elso).

Regarding antimalarial quinolines (Avanzo et al.), the majority of the studies focused on chloroquine and sitamaquine, which showed excellent clinical results. Other quinolines, such as primaquine and amodiaquine, showed great potential for further preclinical studies, whereas ferroquine has been shown to be the least effective antimalarial agent.

The use of a drug delivery system (DDS) to encapsulate and improve the efficacy of leishmanicidal quinolines was discussed (Romero et al.). The particle size, type of DDS, and nature of the polymeric matrix were found to play an important role in the design of micro/nanoformulation. The encapsulation of quinolines is concentrated on polymeric NPs and liposomes. The majority of the studied cases were tested on VL models. NPs based on PLGA and liposomes have emerged as the most convenient DDS for achieving good pharmacokinetic/therapeutic profiles.

The pivotal role of lipophilicity and acidity in the design of leishmanicidal agents to promote drug accumulation in phagolysosomes was analyzed in an article, revealing Log *P*∼4-6 and *pK*_*a2*_ ∼8-9 as optimal parameters for generating highly active and selective quinolines (Romero).

Finally, a full compilation of synthetic strategies and their reactions to access the highly versatile 4-quinoline was discussed (Delgado et al.). These reactions are based on: (i) nucleophilic aromatic substitution via conventional heating, microwave, and ultrasound; (ii) one-pot metal-free or metal-catalyzed inter- and intramolecular cyclization/annulation; (iii) miscellaneous reactions including dehydrogenative amination of dihydroquinolin-4(*1H*)one, amination via Hartwig-Buchwald cross coupling and rearrangement reactions.

In summary, the articles presented in this Research Topic collectively highlight the potential of quinoline as a scaffold for the design of potent and selective leishmanicidal agents. The structure-activity relationships, the role of quinoline substitution, the mechanisms, immune issues, synthetic aspects, the role of nanotechnology, and the physicochemical aspects (lipophilicity and acidity) all contributed significantly to demonstrating the broad potential of quinoline as a privileged scaffold. The insights provided in this Research Topic represent an invaluable resource for researchers and clinicians working toward the development of more effective leishmanicidal agents.
